# Theta and beta oscillatory dynamics in the dentate gyrus reveal a shift in network processing state during cue encounters

**DOI:** 10.3389/fnsys.2015.00096

**Published:** 2015-07-01

**Authors:** Lara M. Rangel, Andrea A. Chiba, Laleh K. Quinn

**Affiliations:** ^1^Cognitive Rhythms Collaborative, Laboratory of Cognitive Neurobiology, CAS Psychology, Boston UniversityBoston, MA, USA; ^2^Department of Cognitive Science, University of California San DiegoLa Jolla, CA, USA

**Keywords:** theta, beta, oscillations, dentate gyrus, hippocampus

## Abstract

The hippocampus is an important structure for learning and memory processes, and has strong rhythmic activity. Although a large amount of research has been dedicated toward understanding the rhythmic activity in the hippocampus during exploratory behaviors, specifically in the theta (5–10 Hz) frequency range, few studies have examined the temporal interplay of theta and other frequencies during the presentation of meaningful cues. We obtained *in vivo* electrophysiological recordings of local field potentials (LFP) in the dentate gyrus (DG) of the hippocampus as rats performed three different associative learning tasks. In each task, cue presentations elicited pronounced decrements in theta amplitude in conjunction with increases in beta (15–30 Hz) amplitude. These changes were often transient but were sustained from the onset of cue encounters until the occurrence of a reward outcome. This oscillatory profile shifted in time to precede cue encounters over the course of the session, and was not present during similar behaviors in the absence of task relevant stimuli. The observed decreases in theta amplitude and increases in beta amplitude in the DG may thus reflect a shift in processing state that occurs when encountering meaningful cues.

## Introduction

Hippocampal networks are characterized by multiple interacting rhythms (Buzsáki and Draguhn, 2004). As indicators of large-scale synchronous activity, oscillations can provide meaningful insight into the temporal dynamics of inputs into the hippocampus, as well as local processing within the structure during events that are important for learning and memory. For example, the theta rhythm, which occurs in frequencies between 5 and 10 Hz, can dominate the hippocampal local field potential during voluntary exploratory activity and REM sleep (O'Keefe and Recce, 1993; Bland and Oddie, 2001; Buzsáki, 2002). Contributions to the generation of the theta rhythm in this region include inputs from the medial septum (Lee et al., 1994; Brandon et al., 2011; Koenig et al., 2011) as well as the intrinsic recurrent connectivity of the region (Kocsis et al., 1999). It is hypothesized that the temporal organization of spiking activity into rhythms such as theta could facilitate the formation of distinct processing states within local circuits, or potentially provide a temporal packaging for information to be optimally read or ignored (Buzsáki, 2010; Kopell et al., 2010).

The hippocampus can also exhibit large amplitude higher frequency oscillations in the beta (15–30 Hz), low gamma (30–60 Hz), high gamma (60–90 Hz), and ripple (180–200 Hz) frequency ranges that could interact with or become more dominant than theta (Martin et al., 2007; Berke et al., 2008; Colgin et al., 2009; Buzsáki, 2010; Sullivan et al., 2011; Igarashi et al., 2014). The prevalence and interaction of theta and higher frequency rhythms during associative tasks can provide insight into how the local processing of information within the hippocampal circuit changes at different stages of an experience to facilitate learning. For example, during olfactory appetitive learning tasks, transient oscillatory bursts in the beta frequency range have been recorded in the dentate gyrus and CA1 subregions of the hippocampus during the sampling of odor cues that dictate subsequent correct behavior. These beta bursts have been shown to demonstrate phase coherence with beta oscillations in the olfactory bulb and the upstream lateral entorhinal cortex, and are correlated with the onset of learning and the formation of ensembles (Kay and Freeman, 1998; Vanderwolf, 2001; Martin et al., 2007; Gourévitch et al., 2010; Igarashi et al., 2014; Rangel and Eichenbaum, 2014). The stimuli and behaviors that induce these transient changes in local processing in the hippocampus have not yet been fully characterized.

We utilized three associative learning tasks to observe changes in theta and beta frequency amplitude across different cue modalities. In one task, *in vivo* electrophysiological recordings were performed in rats trained to run laps along a circular track for a food reward in a reliably rewarded location. We observed a large reduction of theta amplitude and an increase in beta amplitude when rats encountered a conditioned reward location. As theta is correlated with running speed, we examined theta and beta amplitude during random stopping behavior at non-rewarded locations. During random stops, although there was a delayed decrease in theta amplitude, there was no significant increase in beta amplitude. We then systematically investigated theta and beta oscillatory dynamics during two additional tasks in order to identify the behavioral task parameters contributing to the observed changes in amplitude. All behavioral tasks revealed the same dynamics of theta decreases concurrent with beta increases upon cue encounter, lasting throughout the receipt of a reward, suggesting a role for beta in the processing of meaningful cues.

## Results

### Circular track task

**Figure 1:**
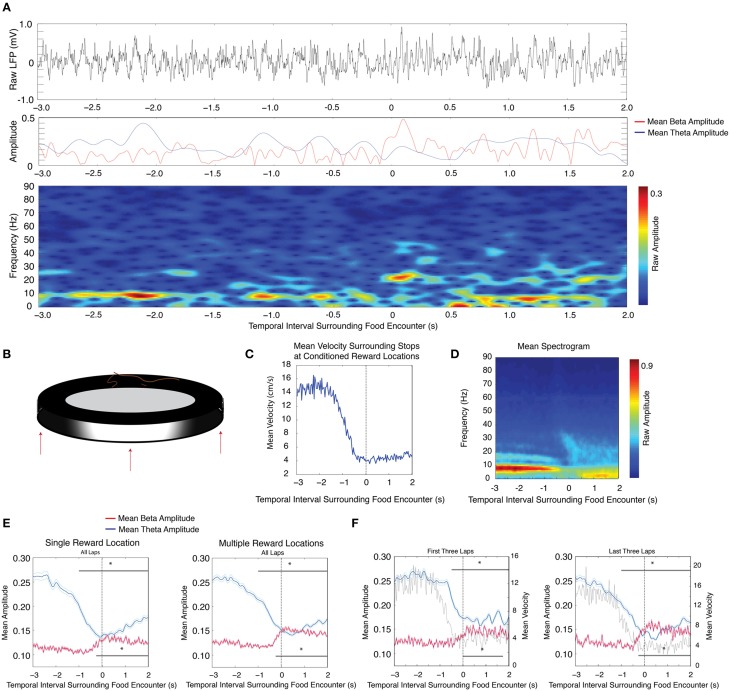
**Decreases in theta (4–12 Hz) amplitude and increases in beta (15–30 Hz) amplitude in response to conditioned reinforcement in a circular track paradigm. (A)** Upper: Raw LFP trace as the rat approaches and stops at the reward location. The zero time point indicates a stop at the reward location. Middle: Instantaneous theta amplitude (blue) or beta amplitude (red) during the same time interval as the raw LFP trace. Lower: Gabor spectrogram during the same time interval as the raw LFP trace. **(B)** Cartoon schematic of the circular track apparatus with up to three example reward locations shown. **(C)** Mean velocity during intervals surrounding stops at conditioned reward locations. **(D)** Mean spectrogram across all sessions and all rats. Large amplitude 16 Hz oscillations prior to stops at conditioned reward locations likely reflect a theta harmonic. **(E)** Mean theta (*blue*) and mean beta (*red*) amplitude (mV) for recording sessions in which there was one (left) or multiple (right) conditioned reward locations. The zero point marks the rat's encounter with a conditioned reward location. The upper gray bar indicates 250 ms bins with significant decreases in theta amplitude and the lower gray bar indicates 250 ms bins with significant increases in beta amplitude. Error bars indicate standard error for theta (*cyan*) and beta (*magenta*) means. **(F)** Same as in **(E)**, for the first three (left) and last three (right) laps of sessions in which there was more than one shift in reward location, with gray traces indicating the mean velocity; significant via Tukey's HSD, ^*^p < 0.05.

Local field potential recordings were acquired from the dentate gyrus granule cell layer of nine rats. These rats successfully learned to complete full laps around a circular track in order to receive a food reward in a reliably rewarded location. After the successful completion of at least 15 laps, the reward location was changed up to three times per session. We calculated the average theta (5–10 Hz) and beta (15–30 Hz) amplitudes during 5 s intervals surrounding stops at conditioned reward locations in steps of 250 ms time bins (See Materials and Methods). In recording sessions in which there was a single conditioned reward location, theta amplitude underwent significant reduction 1 s prior to stopping behavior and maintained reduced amplitude throughout the 2 s examined after the initiation of stopping behavior (Figures 1A,D,E (left panel, *blue*); *n* = 9 rats, 18 sessions, repeated measures ANOVA, d.f. = 12, *F* = 18.54, *p* < 0.00001). In contrast, beta frequency oscillations showed a significant increase in amplitude starting 250 ms prior to stops at conditioned reward locations [Figure 1E (left panel, *red*), repeated measures ANOVA, d.f. = 12, *F* = 11.97, *p* < 0.00001]. In recording sessions in which the reward location was shifted, rats learned to stop at the new conditioned reward locations for food. During these sessions, decreases in theta amplitude [Figures 1A,D,E (right panel, *blue*); *n* = 9 rats, 19 sessions, repeated measures ANOVA, d.f. = 12, *F* = 21.28, *p* < 0.00001] and increases in beta amplitude [Figure 1E (right panel, *red*), repeated measures ANOVA, d.f. = 12, *F* = 19.25, *p* < 0.00001] were also observed across location sites.

We then examined whether these changes in amplitude were modulated during learning the significance of conditioned reward locations. On days in which conditioned reward locations were shifted, we determined whether these changes in amplitude existed during the first three and last three laps at each reward location. During the first three laps at new conditioned reward locations, significant decreases in theta amplitude were observed beginning 500 ms prior to stops as well as throughout stops, whereas significant increases in beta amplitude were only observed during intervals after stops [Figure 1F (left panel, theta *blue*, beta *red*), repeated measures ANOVA, for theta d.f. = 12, *F* = 17.42, *p* < 0.00001, for beta d.f. = 12, *F* = 7.71, *p* < 0.00001]. Analysis of the last three laps from the same sessions showed significant decreases in theta amplitude beginning 1 s prior to stops as well as throughout stops, and significant increases in beta amplitude beginning 250 ms prior to stops and lasting throughout stops [Figure 1F (right panel, theta *blue*, beta *red*), repeated measures ANOVA, for theta d.f. = 12, *F* = 11.67, *p* < 0.00001, for beta d.f. = 12, *F* = 15.1, *p* < 0.00001]. Although a 2-factor repeated measures ANOVA did not reveal an interaction effect on beta amplitude between early and late laps (group) and time relative to stop (repeated measures ANOVA, d.f. = 1, *F* = 1.29, *p* = 0.2229), we performed a planned comparison of the relevant 250 ms interval just prior to the stops at the reward location and observed that beta amplitude increases were significantly higher during the last three laps than during the first three laps (paired *t*-test, d.f. = 18, *p* = 0.04). There was no interaction effect on velocity between early and late laps (group) and time relative to stop [repeated measures ANOVA (d.f. = 12, *F* = 0.75, *p* = 0.706)]. Whereas significant decreases in theta amplitude regularly preceded stops at conditioned reward locations, even during the first three laps at a new reward location, significant increases in beta amplitude were not observed prior to stops during the first three laps. This suggests that beta amplitude increases anticipating the stop are acquired over time.

### Random stops on the circular track

**Figure 2:**
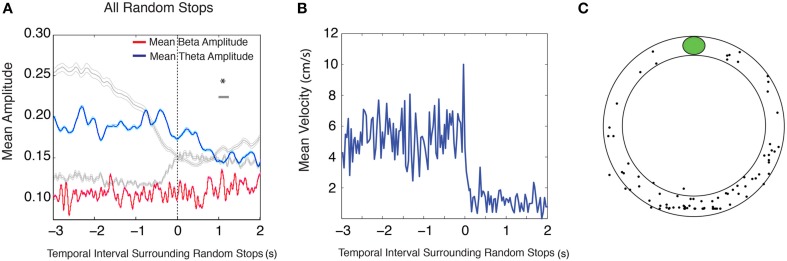
**Random stops on the circular track do not elicit changes in theta and beta amplitude. (A)** Mean theta (*red*) and mean beta (*blue*) amplitude (mV) during intervals surrounding stops longer than 3 s. Gray traces outline the mean theta and mean beta amplitude during stops at conditioned reward locations shown in Figure 1E, right. The zero point indicates the onset of a stop. The upper gray bar indicates 250 ms bins with significant decreases in theta amplitude (*blue*) and the lower gray bar indicates 250 ms bins with significant increases in beta amplitude (*red*). Error bars indicate standard error for theta (*cyan*) and beta (*magenta*) means. **(B)** Mean velocity during intervals surrounding random stops. **(C)** Distribution of random stops with respect to reward location. The plot does not depict the physical location of a stop on the circular track, but rather the location of a stop with respect to a given condition reward location; significant via Tukey's HSD, ^*^p < 0.05.

Occasionally during running in the circular track task, rats would stop at random times and random locations. It is possible that the changes observed in theta and beta amplitudes during conditioned reward paradigms are solely due to stopping behavior (for mean velocity surrounding approach to conditioned reward locations see Figure 1C) and that these amplitude changes are not related to encountering meaningful cues. An analysis of 5 s intervals surrounding random stopping behaviors was performed across stops that were dispersed around the track (for mean velocity surrounding random stops see Figure 2B, for stop locations see Figure 2C, see Materials and Methods for random stop inclusion criteria). This revealed a decrease in theta amplitude after the stop [Figure 2A (*blue*); *n* = 9 rats, 28 sessions and 87 stops, repeated measures ANOVA, d.f. = 12, *F* = 7.87, *p* < 0.00001], and a lack of increase in beta amplitude [Figure 2A (*red*); repeated measures ANOVA, d.f. = 12, *F* = 1.61, *p* = 0.084 for beta], suggesting that late theta amplitude decreases, but not beta amplitude increases are linked to the stopping behavior.

### Conditioned cue reward task

**Figure 3:**
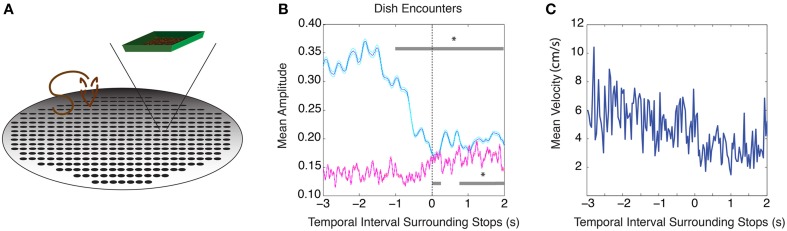
**Conditioned cue paradigm elicits similar changes in theta and beta amplitude. (A)** Cartoon schematic of the task. **(B)** Mean theta (*blue*) and mean beta (*red*) amplitude as the rat encounters the conditioned cue (weigh boat) at the zero time point. The upper gray bar indicates 250 ms bins with significant decreases in theta amplitude (*blue*) and the lower gray bar indicates 250 ms bins with significant increases in beta amplitude (*red*). Error bars indicate standard error for theta (*cyan*) and beta (*magenta*) means. **(C)** Mean velocity during intervals surrounding stops at the conditioned cue; significant via Tukey's HSD, ^*^p < 0.05.

The same 9 rats used in the previous task were trained on a conditioned cue reward task that did not rely on spatial cues. In this task, rats foraged for randomly scattered chocolate sprinkles within a large circular arena, and learned to approach weigh-boats filled with chocolate sprinkles that were presented at three random times and at random locations. As spatial features of an environment were not meaningful cues in this task, it allowed us to test whether changes in theta and beta amplitude could be elicited from non-spatial cues. As in the circular track paradigm, theta amplitude was significantly reduced beginning 1 s prior to weigh boat encounters compared to 2 s prior to stopping at the weigh boat (Figure 3B, *n* = 9 rats, 25 sessions, repeated measures ANOVA, d.f. = 12, *F* = 26.63, *p* < 0.00001, for mean velocity surrounding weigh boat encounters see Figure 5C). Beta frequency oscillations also showed a significant increase in amplitude at the weigh boat, but not before encounters (repeated measures ANOVA, d.f. = 12, *F* = 7.59, *p* < 0.00001).

### Object association task

**Figure 4:**
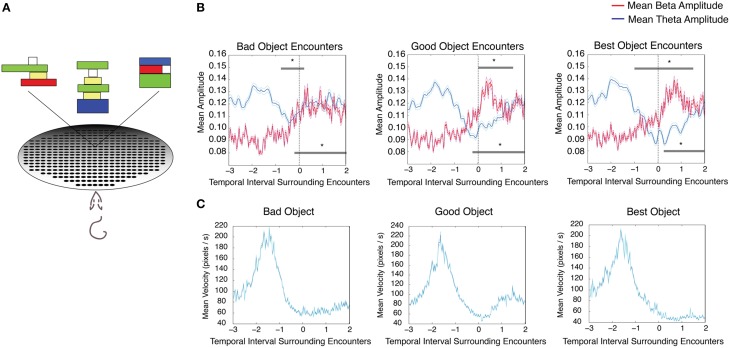
**Changes in theta and beta amplitude during encounters with each object in an object association task. (A)** Cartoon schematic of the task in which rats were trained to approach and push over three Lego objects in order to receive a 100%, (best), 25% (good), or 0.002% quinine (bad) pellet underneath. Each object was conditioned to one of the three pellet types. **(B)** Mean theta (*blue*) and mean beta (*red*) amplitude for encounters with the bad (left), good (middle), and best objects (right). The upper gray bar indicates 250 ms bins with significant decreases in theta amplitude (*blue*) and the lower gray bar indicates 250 ms bins with significant increases in beta amplitude (*red*). Error bars indicate standard error for theta (*cyan*) and beta (*magenta*) means. **(C)** Mean velocity during intervals surrounding object encounters in the object association task; significant via Tukey's HSD, ^*^p < 0.05.

Four rats that were not utilized in the previously described tasks were trained on an object association task. Here the rats learned the associative value of three abstract Lego® objects consistently paired with food pellets of three different values. This task controlled for odor across conditions and contained one aversive (0.002% quinine) as well as two positive outcomes (25% sucrose and 100% sucrose), thus providing a direct test of the role of reward value in modulating theta and beta amplitudes in the absence of olfactory cues. All rats learned to reliably approach and push over objects to receive (or decline) food pellets of different valence underneath (for mean velocity surrounding object encounters, see Figure 4C). Although there was a significant interaction effect on velocity between object type (group) and time relative to stop (repeated measures ANOVA, velocity_Best_v velocity_Good_, d.f. = 12, *F* = 6.24, *p* < 0.00001; velocity_Best_v velocity_Bad_, d.f. = 12, *F* = 2.69, *p* = 0.0015; velocity_Good_v velocity_Bad_, d.f. = 12, *F* = 2.69, *p* = 0.0015), similar theta and beta amplitude changes observed in previous tasks were observed at the rat's encounter with each of the three object types. Significant decreases in theta amplitude and increases in beta amplitude were seen as rats encountered objects associated with 100% sucrose “best” [Figure 4B (*left*), *n* = 4 rats, 31 sessions, repeated measures ANOVA d.f. = 12, *F* = 18.33, *p* < 0.00001 for theta, d.f. = 12, *F* = 8.35, *p* < 0.00001 for beta], 25% sucrose “good” [Figure 4B (*middle*), repeated measures ANOVA d.f. = 12, *F* = 12.88, *p* < 0.00001 for theta, d.f. = 12, *F* = 9.59, *p* < 0.00001 for beta], and 0.002% quinine “bad” pellets [Figure 4B (*right*), repeated measures ANOVA d.f. = 12, *F* = 3.64, *p* < 0.00001 for theta, d.f. = 12, *F* = 7.21, *p* < 0.00001 beta]. There was no interaction effect on beta amplitude between object type (group) and time relative to stop (repeated measures ANOVA, beta_Best_v beta_Good_, d.f. = 12, *F* = 1.06, *p* = 0.392; beta_Best_v beta_Bad_, d.f. = 12, *F* = 0.79, *p* = 0.660; beta_Good_v beta_Bad_, d.f. = 12, *F* = 0.80, *p* = 0.650).

### Current source density analysis in a delayed alternation task

**Figure 5:**
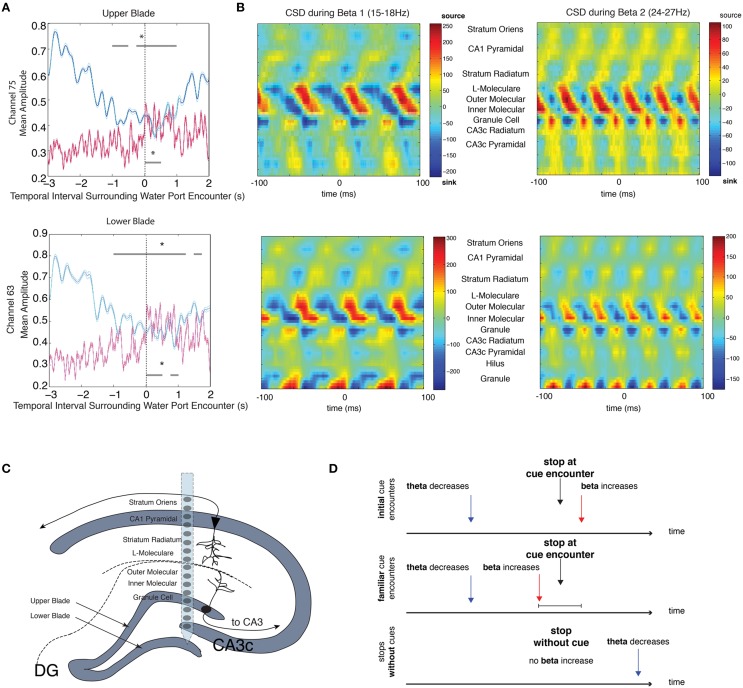
**CSD analysis of beta local field potentials along the D/V Axis. (A)** Mean theta (*blue*) and mean beta (*red*) amplitude for intervals surrounding stops at the water port for a single session (top: upper blade of the granule cell layer, bottom: lower blade of the granule cell layer). The upper gray bar indicates 250 ms bins with significant decreases in theta amplitude (*blue*) and the lower gray bar indicates 250 ms bins with significant increases in beta amplitude (*red*). Error bars indicate standard error for theta (*cyan*) and beta (*magenta*) means. **(B)** Average CSD (current source density) from one rat for the 200 ms surrounding the trough of each beta cycle that met the following conditions: (1) cycle occurred within the 5 s intervals surrounding stops at the water port and (2) beta amplitude was 2 STD above the mean amplitude of the recording session. Given the large range in beta frequencies (15–30 Hz), CSD analysis was performed for two smaller frequency ranges: Beta 1 (15–18 Hz) and Beta 2 (24–27 Hz). **(C)** Schematic of the dendritic and somatic layers of the dentate gyrus, including a cartoon representation of a single silicon probe shank. **(D)** Summary diagram outlining the main findings across experiments. Decreases in theta amplitude are observed prior to stops during initial cue encounters, whereas increases in beta amplitude are observed after stops. Stops at familiar cues elicit similar changes in theta, but increases in beta can be observed prior to stops at familiar cues. When there are no cues present, decreases in theta can be observed after stops, but there are no increases in beta; significant via Tukey's HSD, ^*^p < 0.05.

In order to verify that the observed increases in beta amplitude were due to local circuit dynamics as opposed to volume conductance of rhythmic dynamics from neighboring brain regions, we attempted to identify the source of the beta rhythm. We examined local field potential data from high-density silicon probes spanning the dendritic and somatic layers of the DG, CA3, and CA1 regions of the hippocampus with fixed vertical spacing between electrode sites. This data provided a snapshot of robust rhythmic responses that may occur throughout the hippocampus during associative learning tasks, and the direction of current flow. We attempted to identify source and sink locations in the beta frequency range by calculating the rate of change in current over space (see Materials and Methods, current source density analysis). In the two rats used for this analysis, large decreases in theta amplitude and increases in beta amplitude were observed from channels in the granule cell layer during intervals in which the rats stopped at water ports [Figure 5A, local field potentials (LFP) aligned to upper blade (top) or lower blade (bottom), single session of 26 trials, repeated measures ANOVA, upper blade: d.f. = 12, *F* = 4.96, *p* < 0.00001 for theta, d.f. = 12, *F* = 3.52, *p* = 0.0001 beta, lower blade: d.f. = 12, *F* = 3.92, *p* < 0.00001 for theta and d.f. = 12, *F* = 4.33, *p* < 0.00001 for beta]. Current source density analysis revealed a beta dipole between the molecular (input) layer and granule cell layer of the dentate gyrus in both rats from whom 96-channels were recorded (Figure 5B).

## Discussion

In each of the tasks investigated in this study, decreases in theta amplitude occurred concurrently with increases in beta amplitude during intervals in which cues, such as spatial locations or objects, anticipated an outcome. Each of the tasks aided in disambiguating the task parameters leading to the observed effects. First, although the hippocampus is a structure well characterized for the spatial specificity of its cell activity (O'Keefe and Dostrovsky, 1971), these effects were not solely dependent upon spatial cues, as the conditioned cue task elicited decreases in theta amplitude and increases in beta amplitude at previously unrewarded spatial locations. These effects were also not solely dependent upon olfaction, as the object association task controlled for olfactory cues (See Materials and Methods). Further, these effects were not contingent upon a rewarding outcome, as the object association task contained an object associated with a pellet of negative valence. Finally, the late decrease in theta amplitude and lack of increase in beta amplitude after random stops on the circular track suggest that these changes are not merely the result of stopping behavior. A parameter common to each of these tasks was that a cue, which could be composed of several different modalities including sensation and location, anticipated an outcome (Figure 5D). While we cannot rule out that these oscillatory dynamics might reflect shifts in local circuit processing states driven by behaviors present in each task (for example sniffing, anticipation of reward, or motivation to obtain a reward), the specific temporal dynamics suggest that the processing of meaningful cues engenders the observed theta and beta modulatory pattern.

These oscillatory dynamics, and particularly the switch from theta to beta as the dominant rhythm in the dentate gyrus, suggest that the hippocampus is entering a different processing state, with each rhythm operating under different neural mechanisms. The random stop finding suggests that the decrease in theta is highly correlated with decreasing movement, consistent with previous studies that show theta amplitude is tightly linked to velocity (McFarland et al., 1975; Sławińska and Kasicki, 1998). Notably, decreases in theta are present during random stopping behavior even when there are no statistically significant increases in beta. This finding is supportive of the idea that the theta decrement and beta increase operate as independent processes. However, it could still be the case that the theta decrease is permissive of cue-induced oscillatory patterns such as the observed increase in beta power.

Several questions regarding the etiology of these two signals remain. It is possible from the results of the current source density analysis that the beta rhythm is generated from the perforant path inputs of the entorhinal cortex that project to the outer 2/3 of the molecular layer of the dentate gyrus. The entorhinal cortex is at an ideal intersection of highly processed sensory information in rodents and primates (Suzuki et al., 1997; Young et al., 1997; Furtak et al., 2007), and also receives strong projections from the amygdala and dopaminergic centers (Insausti et al., 1987; Saunders and Rosene, 1988; Stefanacci et al., 1996; Pitkänen et al., 2000). The beta rhythm has been hypothesized to be a facilitator of coordination across brain structures (Kopell et al., 2000; Bibbig et al., 2002; Pinto et al., 2003), and may be ideal for the coordination of information from multiple input structures.

Our findings are consistent with previous studies showing increases in beta frequency oscillations in the hippocampus, olfactory bulb, and entorhinal cortex (which projects to both the hippocampus and olfactory bulb) during olfactory discrimination tasks (Kay and Freeman, 1998; Martin et al., 2007; Gourévitch et al., 2010), as well as increased coherence between the three regions concurrently with the onset of associative learning (Kay and Freeman, 1998; Igarashi et al., 2014). Additionally, increases in beta frequency activity have been observed in the cat hippocampus during the presentation of conditioned stimuli in an eyeblink-conditioning task (Múnera et al., 2001), and in the mouse hippocampus during approaching and pressing a lever in an operant conditioning task (Jurado-Parras et al., 2013), corroborating a role for beta oscillations in associative learning. The location of the beta dipole in our current source density analysis suggests that beta rhythmic activity is generated via perforant path input from the entorhinal cortex (Figure 5C). A recent study has shown that the synaptic strength of perforant synapses in dentate gyrus, CA3, and CA1 are modified over the course of contextual learning, with the largest modifications observed in dentate gyrus (Carretero-Guillén et al., 2015). Our oscillatory findings could thus reflect beta rhythmic activity from the entorhinal cortex to the hippocampus, and particularly the dentate gyrus, when encountering meaningful cues. Alternatively, beta could be a locally emergent rhythm instigated by specific inputs and levels of excitatory drive. In either case, it is possible that the beta rhythm reflects a local processing of information in the dentate gyrus that is distinct from theta epochs, resulting from a dynamic coordination of interacting interneuron and granule cell types. Such changes in processing might then be apparent in the relationship of cell spiking behavior to the local field potential frequencies present. To determine the extent to which theta and beta oscillatory dynamics reflect distinct processing states, future studies will need to compare information processed by cells oscillating in theta rhythmic networks to information processed by cells entrained to beta rhythmic networks.

This study adds to a growing body of literature in which the presence of the beta rhythm is correlated with the presentation of task relevant stimuli. An increase in beta oscillatory activity has been observed during similar task behaviors in associational cortices (Donner et al., 2007, 2009; Buschman et al., 2012), the basal ganglia (Howe et al., 2011; Leventhal et al., 2012), and the basal forebrain (Quinn et al., 2010) across multiple species. Though the functional role of the beta rhythm remains unclear, its presence as a common element across brain regions suggests that it may be a signature of an important organizing mechanism during the processing of meaningful cues.

## Materials and methods

All procedures were performed in accordance with NIH and University of California, San Diego and Rutgers University IACUC guidelines. Seventeen adult male Long-Evans rats were used as subjects. The rats were housed individually and maintained on a 12-h light/dark cycle. They were acclimated to the colony room for 3 days and handled daily for at least 2 weeks prior to beginning the experiment, during which time they were placed on food restriction until they reached 85–90% of *ad libitum* weight. Rats were 3 months old at the time of surgery. Their weights ranged from 300 to 350 g. Water was available at all times. All behavioral testing occurred during the rats' light cycle.

### Circular track training

Nine rats were used in this study. On the first day of circular track training, rats were initially allowed to explore the circular track with randomly spaced ¼ size cereal rewards. After approximately 5 min of random exploration, they began receiving a food reward for successful laps in only one direction along the circular track. On all subsequent days of training, rats received a food reward in a reliably rewarded location only for laps completed in one direction. The food reward would be placed in the rewarded location once rats had traversed half of the circular track, such that the food reward was already present upon stops at the location. On several experiment days, the conditioned food reward location was changed to a new reward location up to three times (Figure 1B). Rats were required to complete at least 15 laps per food reward location. All data analysis was restricted to experiment days after at least 5 days of circular track training.

### Conditioned cue reward training

In this task, the above nine rats were exposed to a circular cheeseboard arena and allowed to explore for randomly spaced chocolate sprinkles for 20 min. At three random time points during exposure to this arena, a large weigh boat filled with chocolate sprinkles was placed in the arena at a random location (Figure 3A). Over many training sessions, rats learned to approach weigh boats immediately after placement in the arena. The weigh boat thus acted as a cue for a large food reward.

### Object association task

Four adult male Long-Evans rats were trained to leave a start box, traverse a 25 inch space, and approach one of three distinct Lego® objects. Rats were allowed to push aside the object in order to obtain a 45 mg food pellet that was consistently paired with the object from a hole underneath (Figure 4A). To ensure that distinct olfactory cues would not bias the response of the rat, each hole of the cheeseboard apparatus was baited with six pellets of each different type that were unattainable due to a protective screen, completely overwhelming the olfactory cue from a single pellet. Each object was paired to one of three differently valued pellets (100% sucrose, 25% sucrose, or 0.002% quinine, Noyes/Research Diets) that rested on top of the protective screen, separating it from the distractor pellets. Consumption tests indicated that the rats showed clear preference for the 100% sucrose over the 25% sucrose pellets as well as a preference for either sucrose pellet over the quinine pellet (data not shown). In order to equate object approach behaviors across the different object-reward association types, rats were trained to approach objects regardless of the pellet type underneath, but could decline to push over objects associated with aversive pellets as a no-go response. Rats were given a total of 6 sessions (one session per day) with a given set of objects, and presented with each of the three object-reward pairing types 10 times per session. Over the course of the first session, rats began to demonstrate no-go responses for the aversive 0.002% quinine object (data not shown). In a previous study, each rat was also tested at the end of the experiment for olfactory cueing by placing the wrong pellet under the object and testing choice behavior. The rats consistently chose on the basis of object association as opposed to pellet type (Quinn et al., 2010). All recordings were acquired during sessions 4–6 of a given object set.

### Microdrive implantation surgery and neural recordings

A microdrive consisting of 3–4 tetrodes of 17 μm platinum iridium wire was surgically implanted using stereotaxic procedures (from bregma A/P: −4.0, M/L: +2.2 mm, D/V: −2.2 mm) and lowered into the granule cell layer of the dentate gyrus (D/V: ~2.7 mm) until the appearance of place cell single units, “dentate spikes,” and complex high frequency (15–90 Hz) local field potential activity. Verification of tetrode locations in dentate gyrus were shown previously (Rangel et al., 2014). Neural recordings of LFP were obtained as previously described (Quinn et al., 2010).

### Analysis of theta and beta amplitude surrounding conditioned location/object encounters

To isolate theta and beta amplitudes surrounding food encounter on the circular track, a third order Butterworth filter (Rubino et al., 2006) was first used to bandpass filter the local field potential (LFP) between 5–10 or 15–30 Hz, respectively. These frequency ranges were chosen to be within the observed range of frequencies present in the average spectrogram during circular track running upon conditioned reward location encounter (Figure 1D). The instantaneous phase and amplitude were then calculated from the Hilbert transform of the filtered signal (Buzsáki et al., 2003; Rubino et al., 2006). The LFPs were then aligned according to isolated intervals (3 s prior to 2 s after food encounters for all laps), averaged for each session, and then compared for every recording. We applied a Gabor transform (Leventhal et al., 2012) to the local field potential signal to create single trial spectrograms (Figure 1A, bottom). The average spectrograms (Figure 1D) were created by first averaging across trials within a session and then across all sessions.

To isolate theta and beta amplitudes surrounding food dish encounters on the cheeseboard arena, the same analysis described above was performed for the 5 s surrounding the start of weigh dish encounters. The mean theta and mean beta amplitudes of all dish encounters were calculated for every recording session. Similarly, the same analysis described above was performed for the 5 s surrounding initial object encounter for each of the three object types in the object association task (best, good, and bad objects). The mean theta and mean beta amplitudes of all trials were calculated for each object type for a given recording session.

For each of the three tasks, mean theta and beta amplitudes during a baseline period were compared to mean theta and beta amplitudes in 250 ms intervals starting at 1 s prior to the time of location/object encounter and lasting up to 2 s after the encounter. A 250 ms interval starting 2 s prior to location/object encounter was chosen as a pre-encounter baseline for comparison as this is typically an epoch of stereotyped running behavior before the presentation of cues. Time intervals beyond 2 s after location/object encounters were not examined, as the rat behavior becomes variable beyond 2 s. Intervals were compared using a repeated measures ANOVA to observe overall differences in conditions. To examine amplitude differences across intervals of 250 ms, subsequent pair-wise comparisons were performed and adjusted for multiple comparisons using a Tukey's honestly significant difference criterion.

### Analysis of theta and beta amplitude surrounding random stops

To further characterize the behavioral significance of changes in theta and beta amplitude at conditioned food and object encounters in these experiments, theta and beta amplitudes during intervals surrounding random stops in the circular track task were examined as described in the location/object encounter methods. To test whether theta and beta amplitude changes were due to simple stopping behaviors, we examined whether these changes occurred during intervals surrounding random stops on the circular track. Random stops on the circular track were defined as stops made at least 30 cm away from the conditioned reward location for durations of at least 3 s. The positions of the random stops were plotted to verify that they did not represent a stereotyped stopping behavior on the circular track (Figure 2C).

### Current source density analysis

To determine the source of the beta rhythm, current source density analysis was performed on data from 2 rats containing 96-channel probes with local field potential recordings fixed spatially. Probes consisted of 6 shanks with lateral spacing of 300 μm apart. Each shank contained 16 recording sites that were spaced 100 μm apart along the dorsal/ventral axis of the hippocampus (Montgomery et al., 2009). Rats containing these probes performed a delayed alternation task in which they traveled down a common arm from a start chamber to the far end of a circular track and alternated between choosing left and right return arms to the start chamber. Single water ports placed along the left and right return arms provided a water reward for correct choices. As described previously, the average theta and beta (15–18 and 24–27 Hz) amplitude signals during 5 s intervals surrounding water port encounters were calculated. For current source density (CSD) analysis, the second spatial derivative was calculated for all channels using the two channels above and below each channel of interest. 200 ms intervals were identified and isolated that surrounded the trough of each beta cycle in which (1) beta amplitude in the dentate gyrus was 2 standard deviations above the mean for the recording and (2) the beta cycle occurred within the 5 s surrounding stops at the water port. The CSD was then aligned for each beta trough and averaged to reveal any robust changes in current over space. Two narrow frequency ranges of beta were chosen given the large range of frequencies between 15 and 30 Hz and in order to separately assess the source of a low (15–18 Hz) and high (24–27 Hz) beta that have been shown previously to be functionally distinct in other brain regions (Kramer et al., 2008; Roopun et al., 2008).

### Conflict of interest statement

The authors declare that the research was conducted in the absence of any commercial or financial relationships that could be construed as a potential conflict of interest.
